# New small‐molecule compound Hu‐17 inhibits estrogen biosynthesis by aromatase in human ovarian granulosa cancer cells

**DOI:** 10.1002/cam4.3492

**Published:** 2020-10-01

**Authors:** Yang Xi, Jiansheng Liu, Haiwei Wang, Shang Li, Yanghua Yi, Yanzhi Du

**Affiliations:** ^1^ Center for Reproductive Medicine Ren Ji Hospital School of Medicine Shanghai Jiao Tong University Shanghai China; ^2^ Shanghai Key Laboratory for Assisted Reproduction and Reproductive Genetics Shanghai China; ^3^ Central Laboratory Shanghai Xuhui Central Hospital Zhongshan‐Xuhui Hospital Fudan University Shanghai China; ^4^ Institute of Health Sciences School of Medicine (SJTUSM)/Shanghai Institutes for Biological Sciences Chinese Academy of Sciences Shanghai Jiao Tong University Shanghai China; ^5^ Research Center for Marine Drugs School of Pharmacy Second Military Medical University Shanghai China

**Keywords:** apoptosis, *CYP19A1*, estrogen, ovarian granulosa cancer

## Abstract

Estrogen‐dependent cancers (breast, endometrial, and ovarian) are among the leading causes of morbidity and mortality in women worldwide. Aromatase is the main enzyme that catalyzes the biosynthesis of estrogen, which drives proliferation, and antiestrogens can inhibit the growth of these estrogen‐dependent cancers. Hu‐17, an aromatase inhibitor, is a novel small‐molecule compound that suppresses viability of and promotes apoptosis in ovarian cancer cells. Therefore, this study aimed to predict targets of Hu‐17 and assess its intracellular signaling in ovarian cancer cells. Using the Similarity Ensemble Approach software to predict the potential mechanism of Hu‐17 and combining phospho‐proteome arrays with western blot analysis, we observed that Hu‐17 could inhibit the ERK pathway, resulting in reduced estrogen synthesis in KGN cells, a cell line derived from a patient with invasive ovarian granulosa cell carcinoma. Hu‐17 reduced the expression of *CYP19A1* mRNA, responsible for producing aromatase, by suppressing the phosphorylation of cAMP response element binding‐1. Hu‐17 also accelerated aromatase protein degradation but had no effect on aromatase activity. Therefore, Hu‐17 could serve as a potential treatment for estrogen‐dependent cancers albeit further investigation is warranted.

## Graphical abstract

The present study suggests that Hu‐17 inhibits estrogen biosynthesis by decreasing aromatase expression and destabilizing aromatase protein to inhibit cell proliferation and induce cell apoptosis in KGN cells, and it warrants further investigation as potential treatment for estrogen‐dependent cancers.

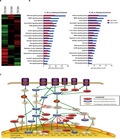

## 1 BACKGROUND

Gynecological malignancies such as ovarian, breast, and endometrial cancers are the leading cause of cancer‐related deaths in women.^1^ Ovarian cancer is the second most commonly diagnosed cancer in women worldwide,^2^ and the mortality rate is worse than other reproductive cancers because of difficulty in early diagnosis.^3^, ^4^ Surgery followed by chemotherapy is currently the standard therapy for ovarian cancers.^5^ Platinum‐based drugs (e.g., cisplatin and carboplatin) and paclitaxel are traditional first‐line therapies.^6^, ^7^ Although ovarian cancer diagnosed in the early stage may be treated successfully, as the disease progresses, most advanced‐stage patients develop resistance to first‐line drugs.^8^ Therefore, there is an urgent need to explore more therapeutic agents to overcome drug resistance.

Estrogens play a pivotal role in the development of gynecological cancers.^9^, ^10^ Therefore, a new strategy developed to treat estrogen‐mediated carcinogenesis involves inhibiting cell proliferation by reducing the estrogen levels.^11^, ^12^, ^13^ There are two major therapeutic approaches to disrupt estrogen function. The first is to block estrogen binding to the estrogen receptor (ER) via ER antagonists. The second is to reduce estrogen biosynthesis with aromatase inhibitors.^13^, ^14^, ^15^ Aromatase, encoded by the *CYP19A1* gene, is a rate‐limiting enzyme that catalyzes estrogen biosynthesis and is highly expressed in the placenta, breast, and granulosa cells of ovarian follicles.^9^, ^16^, ^17^ Multiple studies indicate that aromatase inhibitors are better tolerated than ER antagonists, in addition to being less toxic and highly effective in curing estrogen‐dependent cancer.^18^, ^19^ There are two types of aromatase inhibitors, steroidal (e.g., exemestane) and nonsteroidal (e.g., letrozole) that can be used to treat estrogen‐dependent cancers in postmenopausal women.^20^, ^21^, ^22^ However, the inhibition of aromatase results in an increased risk of osteoporosis and cardiovascular disease.^23^, ^24^, ^25^ Therefore, novel aromatase inhibitors with greater clinical efficacy and fewer side effects are needed.

Phytolaccaesculenta (known as *shanglu* in China) is an important traditional Chinese medicine, and a decoction of its root is used to treat inflammation‐related conditions.^26^ Hu‐17, a novel synthetic compound, was derived from the root of phytolaccaesculenta. We found that Hu‐17 can strongly inhibit the proliferation of cells and promote apoptosis in ovarian epithelial carcinoma cell lines and animal models. It was authorized in 2018 (China patent number: ZL201510256415.X). However, the effect of Hu‐17 on ovarian granulosa cell carcinoma is not clear.

Similarity Ensemble Approach (SEA) is a computational strategy that use chemical similarity among ligands organized by their targets to calculate similarities and predict drug off‐target or targeted activities.^27^, ^28^, ^29^ In this study, we used SEA to predict drug targets of Hu‐17 and assess its intracellular signaling in a steroidogenic human ovarian granulosa‐like tumor KGN cell line treated with Hu‐17.

## 2 MATERIALS AND METHODS

### 2.1 Materials

**FIGURE 1:**
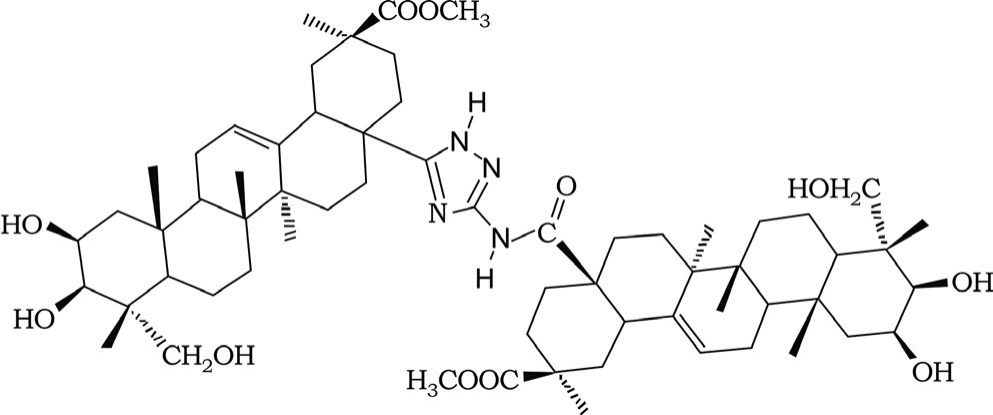
Chemical structure of Hu‐17

Forskolin, exemestane, formestane, cisplatin, and PD98059 were purchased from Sigma Chemical Co. MG132, Z‐VAD‐FMK and cycloheximide were purchased from Selleckchem. Paclitaxel was kindly provided by the Shanghai Key Laboratory of Gynecologic Oncology, Ren Ji Hospital. Hu‐17 was synthesized by the laboratory of Professor Yanghua Yi, Second Military Medical University. Hu‐17 (molecular weight 1084 Da; structure in Figure 1) was stored at −20°C as a stock solution (20 mmol/L) in dimethyl sulfoxide.

### 2.2 Cell culture

Human granulosa cells (hGCs) were collected from patients with ovarian hyperstimulation syndrome (OHSS) and without OHSS undergoing their first in vitro fertilization/intracytoplasmic sperm injection cycle at the Center for Reproductive Medicine, Ren Ji Hospital. All participants provided written informed consent to participate in this study. The isolation protocol for hGCs was performed as described previously.^30^ KGN, a steroidogenic human ovarian granulosa‐like tumor cell line, was kindly provided by the Shandong University, China. MCF‐7 and SUM‐159 cells were purchased from Cell Bank, Chinese Academy of Sciences. The hGCs and KGN cells were maintained in phenol red‐free DMEM/F12 supplemented with 10% charcoal‐stripped fetal bovine serum (FBS). MCF‐7 and SUM‐159 cells were cultured separately in DMEM and F12 supplemented with 10% FBS at 37°C and 5% CO_2_. All media and FBS were purchased from Gibco.

### 2.3 Transfection

KGN cells were transiently transfected with synthetic siRNAs (Gene Pharma) using the Lipofectamine RNAi‐MAX transfection kit (Invitrogen). The nucleotide sequences of *CYP19A1* siRNA was 5′‐GUGGAAUUAUGAGGGCACATT‐3′. Transfection was performed according to the manufacturer's protocol.

### 2.4 Measurement of intracellular cAMP concentration

The intracellular cAMP level in KGN cells was measured using an EIA kit (Cayman, Ann Arbor, MI, USA) after treatment with Hu‐17 (1.5 µmol/L) or forskolin (50 mmol/L) in serum‐free DMEM in the presence of the phosphodiesterase inhibitor 3‐isobutyl‐1‐methyl xanthine (500 mmol/L, Sigma). The assay was performed as described previously.^31^

### 2.5 In vitro aromatase activity assay

The CYP19/MFC High Throughput Inhibitor Screening kit and Baculovirus‐infected insect cell‐recombinant human CYP19 (with oxidoreductase) were purchased from BD Biosciences (Gentest). The in vitro activity of aromatase was determined by measuring the conversion rate of a fluorometric substrate to its fluorescent metabolite. Experimental procedures were consistent with the manufacturer's protocols.

### 2.6 Cell viability and morphological changes

Cell viability was measured using the MTT assay (Sangon Biotech). The assay was performed as described previously.^32^ The morphological changes in KGN cells treated with Hu‐17, CHX, or MG132 were visualized using an inverted microscope connected to a digital camera (Carl Zeiss).

### 2.7 Cell apoptosis

Apoptosis was assessed with Annexin V‐FITC and PI double staining kits (BD Falcon^TM^) by flow cytometry. Samples were subjected to FACS‐Calibur analysis (BD Falcon^TM^) as described previously.^33^

### 2.8 Extraction of RNA and real‐time polymerase chain reaction

*CYP19A1* and cAMP response element binding‐1 (*CREB*‐*1*) mRNA expressions were quantified using a total RNA isolation kit (FOREGENE, Chengdu) and SYBR^®^ Premix Ex TaqTM (TaKaRa) with the following primers (Sangon): **β‐actin**, forward 5′‐GGGAAATCGTGCGTGACATTAAG‐3′ and reverse 5′‐TGTGTTGGCGTACAGGTCTTTG‐3′; ***CYP19A1***, forward 5′‐TGGCTACCCAGTGAAAAAGG‐3′ and reverse 5′‐CCATGGCGATGTACTTTCCT‐3′; ***CREB*‐1**, forward 5′‐CCAAACTAGCAGTGGGCAGT‐3′ and reverse 5’‐CCCCATCCGTACCATTGTT‐3’.

### 2.9 Extraction of protein and western blotting

Protein extraction and western blotting were performed as described previously.^34^ The following primary antibodies were used: anti‐AROM was purchased from Abcam; anti‐CREB‐1, anti‐P90RSK, anti‐pP90RSK, anti‐ERK, anti‐pERK, anti‐MEK, anti‐pMEK, anti‐EGFR, and anti‐c‐Raf were purchased from Cell Signaling Technology; and anti‐AKT, anti‐pAKT, anti‐P38, anti‐pP38, and β‐actin were purchased from Santa Cruz Biotechnology. Secondary antibodies were also purchased from Cell Signaling Technology. A chemiluminescent detection system (Thermo Fisher Scientific, Carlsbad, CA, USA) was used to detect bands with peroxidase activity.

### 2.10 Phosphoprotein profiling with the Phospho Explorer antibody microarray

The Phospho Explorer Antibody Microarray (PEX100) was designed and manufactured by Full Moon BioSystems Inc. Whole‐cell lysates isolated from KGN cells cultured with 1.5 µmol/L Hu‐17 for 0, 3, 6 h were collected in a Protein Extraction Buffer (Full Moon BioSystems Inc.). The array consisted of 1318 phospho‐specific antibodies; detailed information about the array can be found at https://www.fullmoonbio.com/product/phospho‐explorer‐antibody‐array. The antibody array experiment and analysis were performed according to an established protocol.^35^

### 2.11 Prediction of Hu‐17‐target interactions using SEA

The SEA software was used to predict molecular targets for each phytochemical and was standardized as previously described.^27^ Briefly, SEA uses the chemical similarity of a bait molecule against a set of ligands from the ChEMBL database (https://www.ebi.ac.uk/chembl/) annotated to a target to predict whether the bait molecule will modulate that target. An E‐value is derived from a statistical model that represents the probability of observing this raw score by random chance alone. A smaller E‐value indicates stronger overall chemical structural similarity between two sets of compounds. The Tanimoto coefficient, which represents the similarity between any two molecular fingerprints, on a 0.0 (completely dissimilar) to 1.0 (completely similar) scale, was calculated.

### 2.12 Steroid assays

Testosterone (Sigma Chemical Co.) at a concentration of 10^−7 ^ng/mL was added as a substrate for estrogen synthesis in KGN cells. After treatment with Hu‐17 for 24 h, the levels of estrogen were measured using ECLIA (Roche), according to the manufacturer's instructions.

### 2.13 Statistical analysis

Data were compared by either one‐way ANOVA or the *t* test by Duncan's multiple‐range tests. *p* values < 0.05 were considered to be statistically significant. Statistics were performed using GraphPad Prism 5 (GraphPad, San Diego, CA, USA) and the levels of significance are indicated in the figure legends.

## 3 RESULTS

### 3.1 Hu‐17 inhibits cell proliferation and induces cell apoptosis in KGN cells

**FIGURE 2:**
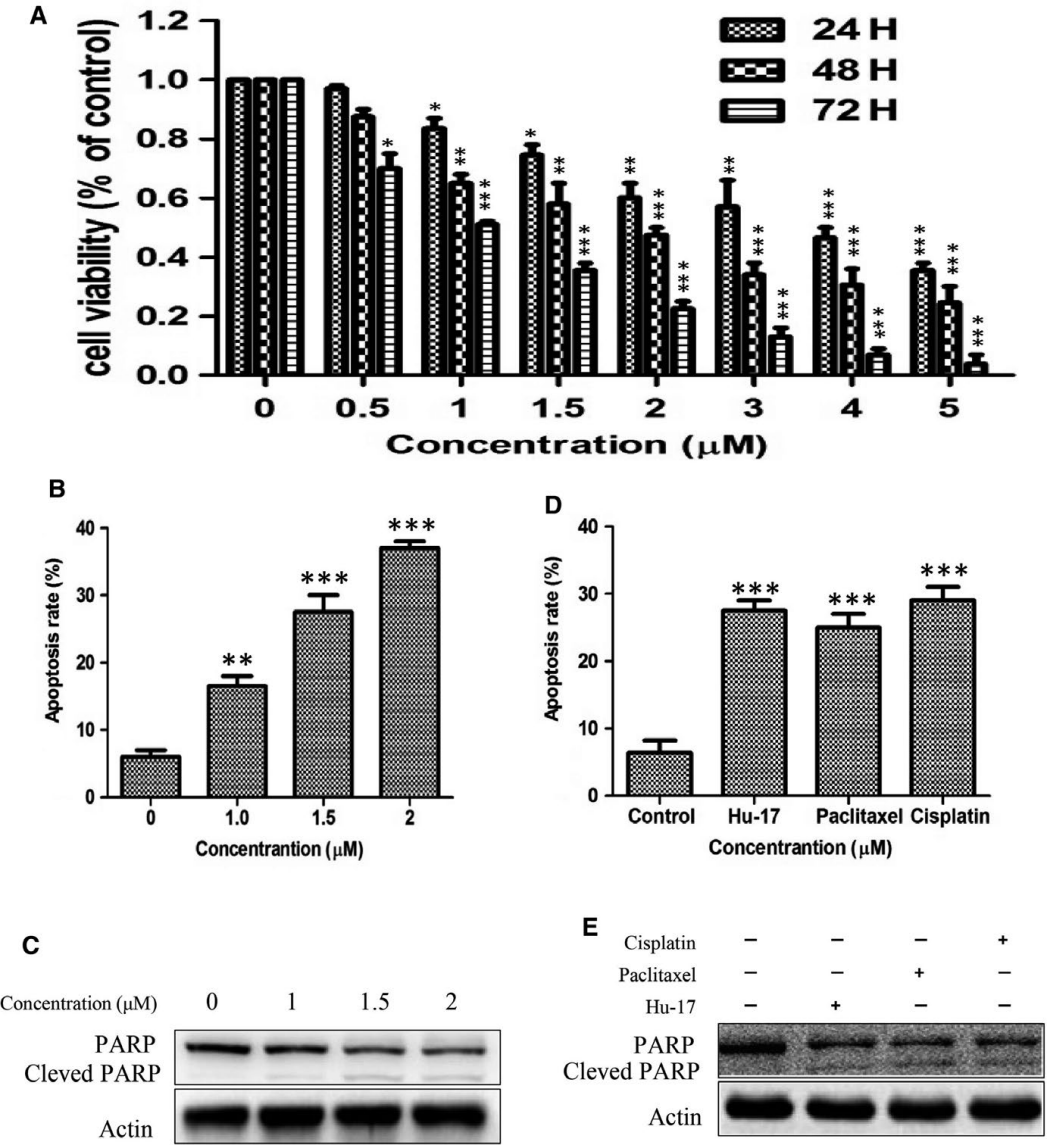
Hu‐17 inhibited cell proliferation and induced cell apoptosis in KGN cells. (A) Cell viability in KGN cells under Hu‐17 (0‐5 μmol/L) treatment for 24, 48 and 72 h assessed by MTT analysis. (B) Induction of apoptosis in KGN cells with the indicated treatments for 24 h, as evaluated through Annexin V‐specific antibody and propidium iodide (PI) staining, and followed by flow cytometry analysis. (C) Western blot analysis of PARP and cleaved‐PARP in KGN cells treated with Hu‐17 at the indicated concentration (n = 3). (D) Induction of apoptosis in KGN cells by 1.5 μmol/L Hu‐17, 15 μmol/L Cisplatin and 100 μmol/L Paclitaxel treatment for 24 h. (E) Western blot analysis of PARP and cleaved‐PARP in KGN cells under the indicated treatments for 24 h (n = 3). All experiments were repeated on three separate occasions, n = 3 means 3 independent experiments. Data are presented as the mean ±SEM (A, B, D). Images represent Western blots (C and E). Statistical analyses were performed using ANOVA (A, B and D). *p < 0.05, **p < 0.01, ***p < 0.001 vs control (0)

Previously, we found that Hu‐17 strongly inhibited ovarian epithelial carcinoma cell proliferation and promoted apoptosis in several ovarian epithelial carcinoma cell lines (including A2780, SKOV3, and HO8910) and mouse models (China patent number: ZL201510256415.X). However, whether Hu‐17 can affect ovarian granulosa cell carcinoma is not clear. Therefore, in order to investigate the role of Hu‐17 on ovarian granulosa cell carcinoma, we choose KGN, a human ovarian granulosa‐like tumor cell line, as a study mode in this study. The survival of KGN cells was assessed using the MTT assay after exposure to a series of doses of Hu‐17 for 24, 48, and 72 h (Figure 2A). Hu‐17 decreased the survival of KGN cells in a both dose‐ and time‐ dependent manners. After treatment with Hu‐17 (0, 1, 1.5, and 2 μmol/L) for 24 h, the proportion of apoptotic cells was 6.2%, 16.5%, 27.6%, and 36.8%, respectively (Figure 2B), indicating that Hu‐17 induced apoptosis in KGN cells in a dose–dependent manner. PARP‐1 cleavage is an early molecular marker of apoptosis. Treatment with 1.5 and 2 μmol/L Hu‐17 for 24 h resulted in considerable PARP‐1 proteolytic cleavage (Figure 2C). We also treated cells with cisplatin and paclitaxel, two chemotherapy drugs currently used in the clinical setting, for comparison with Hu‐17. The proportion of apoptotic cells following treatment with 1.5 μmol/L Hu‐17, 100 μmol/L paclitaxel, and 15 μmol/L cisplatin were similar (27.5%, 25.2%, and 29.1%, respectively) (Figure 2D). Hu‐17, cisplatin, and paclitaxel also increased the abundance of cleaved–PARP‐1 (Figure 2E). These data suggested that Hu‐17 could inhibit proliferation and induce apoptosis in KGN cells.

### 3.2 Predicting potential targets of Hu‐17 using SEA

To gain insights into the mechanism of Hu‐17, we used SEA to identify potential targets. SEA predicted that Hu‐17 might affect hormone signaling, especially estrogen synthesis, by targeting AR, ER, PR, and CYP19A1 in human cells (Table 1). SEA also predicted that Hu‐17 had the potential to affect protein tyrosine phosphatases, including PTPN2, PTPN1, and PTPN6 (Table 1).

**TABLE 1:** Hu‐17 drug‐target prediction by SEA

	Target ID	E‐value	Max Tc	Target name	Target description
1	sp_P04278	3.28 × 10^−43^	0.6627	SHBG_HUMAN	Testis‐specificandrogen‐binding protein
2	sp_Q8NG68	2.66 × 10^−42^	0.5776	TTL_HUMAN	Tubulin‐‐tyrosine ligase
3	sp_P17252	1.62 × 10^−41^	0.5683	KPCA_HUMAN	Protein kinase C alpha
4	P17706	2.48 × 10^−34^	0.3556	PTN2_HUMAN	T‐cell protein‐tyrosine phosphatase
5	sp_P23415	8.84 × 10^−30^	0.5527	GLRA1_HUMAN	Glycine receptor subunit alpha−1
6	sp_P36873	3.85 × 10^−28^	0.5746	PP1G_HUMAN	Serine/threonine protein phosphatase PP1‐gamma
7	sp_P11473	4.43 × 10^−27^	0.5841	VDR_HUMAN	Vitamin D receptor
8	sp_P80365	6.92 × 10^−27^	0.7182	DHI2_HUMAN	11‐beta‐hydroxysteroid dehydrogenase 2
9	sp_P11511	8.88 × 10^−27^	0.6667	CP19A_HUMAN	Cytochrome P450 19A1
10	P80365	1.03 × 10^−22^	0.3238	DHI2_HUMAN	11‐beta‐hydroxysteroid dehydrogenase 2
11	sp_P06746	1.70 × 10^−17^	0.7763	DPOLB_HUMAN	DNA polymerase beta
12	P18031	2.50 × 10^−17^	0.3953	PTN1_HUMAN	Protein‐tyrosine phosphatase 1B
13	P29350	1.55 × 10^−15^	0.2881	PTN6_HUMAN	Protein‐tyrosine phosphatase 1C
14	sp_P05093	3.66 × 10^−14^	0.7143	CP17A_HUMAN	Cytochrome P450 17A1
15	sp_P43088	1.37 × 10^−13^	0.5833	PF2R_HUMAN	Prostanoid FP receptor
16	sp_P04035	7.86 × 10^−13^	0.6847	HMDH_HUMAN	HMG‐CoA reductase
17	sp_P17706	1.22 × 10^−12^	0.7882	PTN2_HUMAN	T‐cell protein‐tyrosine phosphatase
18	sp_P08235	3.84 × 10^−12^	0.5856	MCR_HUMAN	Mineralocorticoid receptor
19	sp_Q05655	9.17 × 10^−12^	0.5645	KPCD_HUMAN	Protein kinase C delta
20	sp_O60218	9.70 × 10^−12^	0.8816	AK1BA_HUMAN	Aldo‐keto reductase family 1 member B10
21	sp_P09884	2.53 × 10^−11^	0.5932	DPOLA_HUMAN	DNA polymerase alpha subunit
22	sp_P10275	2.00 × 10^−10^	0.6944	ANDR_HUMAN	Androgen Receptor
23	sp_P18031	3.67 × 10^−10^	0.8684	PTN1_HUMAN	Protein‐tyrosine phosphatase 1B
24	Q9NZK7	4.10 × 10^−10^	0.3191	PA2GE_HUMAN	Group IIE secretory phospholipase A2
25	pf_2094114	4.61 × 10^−10^	0.6256	CHEMBL2094114	Estrogen receptor
26	Q9BZM2	5.38 × 10^−9^	0.3191	PA2GF_HUMAN	Group IIF secretory phospholipase A2
27	sp_P43116	1.32 × 10^−8^	0.4919	PE2R2_HUMAN	Prostanoid EP2 receptor
28	sp_P50579	1.57 × 10^−8^	0.5561	MAP2_HUMAN	Methionine aminopeptidase 2
29	sp_P16662	3.75 × 10^−8^	0.5432	UD2B7_HUMAN	UDP‐glucuronosyltransferase 2B7
30	sp_P30304	3.81 × 10^−8^	0.5759	MPIP1_HUMAN	Dual specificity phosphatase Cdc25A
31	sp_P11413	4.18 × 10^−8^	0.6024	G6PD_HUMAN	Glucose−6‐phosphate 1‐dehydrogenase
32	Q9UNK4	1.73 × 10^−7^	0.3191	PA2GD_HUMAN	Group IID secretory phospholipase A2
33	sp_P11388	2.37 × 10^−7^	0.7733	TOP2A_HUMAN	DNA topoisomerase II alpha
34	sp_P05129	8.24 × 10^−7^	0.5645	KPCG_HUMAN	Protein kinase C gamma
35	sp_P05412	1.29 × 10^−6^	0.5697	JUN_HUMAN	Proto‐oncogene c‐JUN
36	Q99895	1.68 × 10^−6^	0.2963	CTRC_HUMAN	Chymotrypsin C
37	sp_Q92731	1.87 × 10^−6^	0.6506	ESR2_HUMAN	Estrogen receptor beta
38	sp_Q99895	2.31 × 10^−6^	0.6600	CTRC_HUMAN	Chymotrypsin C
39	sp_Q13133	3.28 × 10^−6^	0.7261	NR1H3_HUMAN	LXR‐alpha
40	sp_P06401	6.29 × 10^−6^	0.6098	PRGR_HUMAN	Progesterone receptor

### 3.3 Hu‐17 inhibits estrogen synthesis and aromatase expression in KGN cells

**FIGURE 3:**
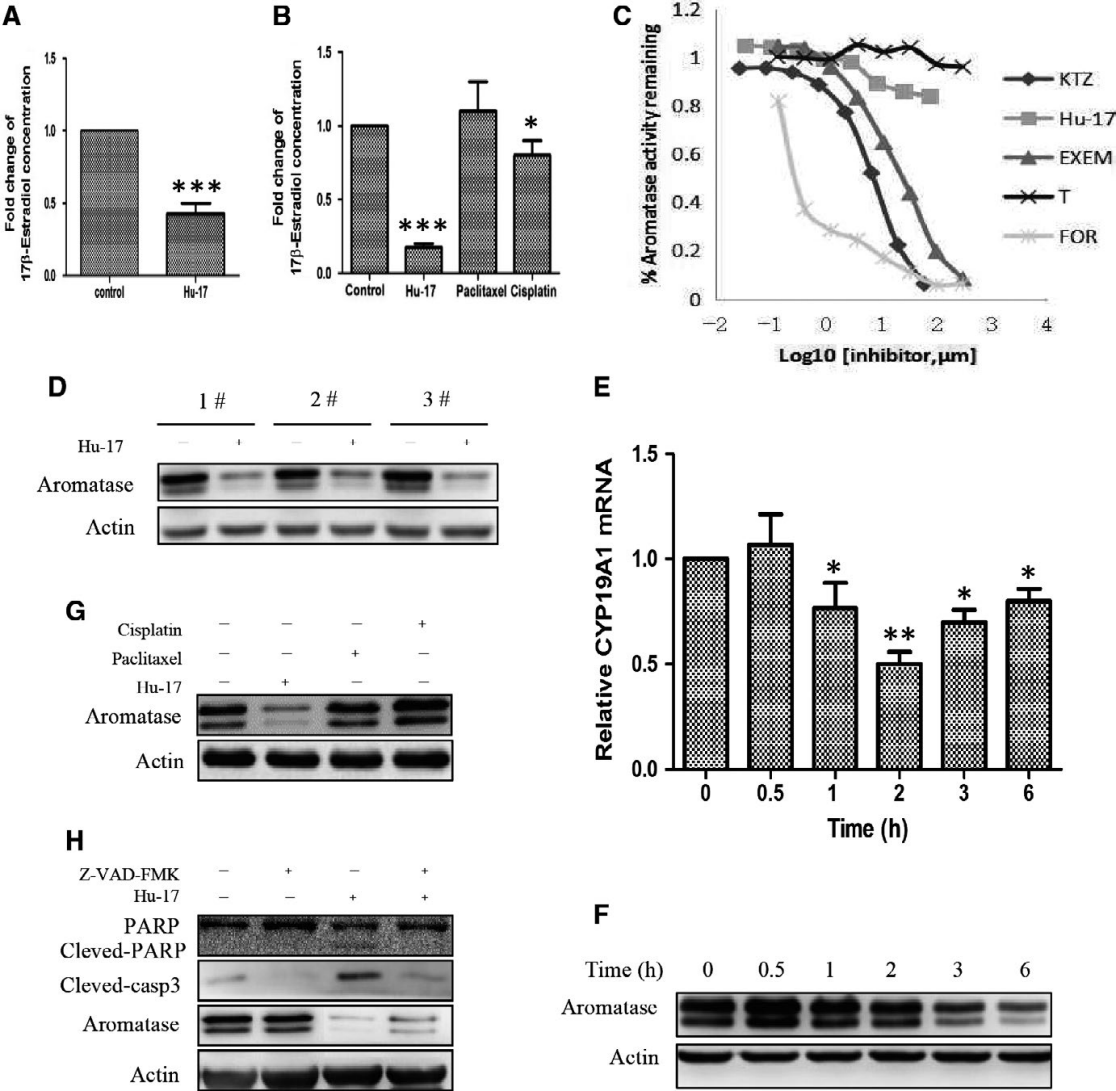
Hu‐17 inhibited estrogen synthesis and aromatase expression in KGN cells. (A) Inhibition of 17β‐estradiol synthesis in primary human ovarian granulosa cells treated with 1.5 μmol/L Hu‐17 for 24 h (n = 3). (B) Effects of Hu‐17 (1.5 μmol/L, 24 h), Cisplatin (15 μmol/L, 24 h) and Paclitaxel (100 μmol/L, 24 h) on 17β‐estradiol synthesis in KGN cells (n = 3). C, The effect of Hu‐17 on the aromatase activity. Curves represent percent aromatase activity remaining in the presence of a range of concentrations of Hu‐17 (0.034‐75 μmol/L), Exemestane (0.137‐300 μmol/L), Ketoconazole (0.027‐60 μmol/L), Testosterone (0.137‐300 μmol/L) and Formestane (0.137‐300 μmol/L) (n = 3). (D) Western blot analysis of aromatase in primary human ovarian granulosa cells treated with 1.5 μmol/L Hu‐17 for 24 h (n = 3). (E and F) *CYP19A1* mRNA expression (E) and aromatase protein abundance (F) in KGN cells treated with 1.5 μmol/L Hu‐17 for 0.5, 1, 2, 3, and 6 h respectively (n = 3). (G) Western blot analysis of aromatase abundance in KGN cells treated with 1.5 μmol/L Hu‐17, 15 μmol/L Cisplatin and 100 μmol/L Paclitaxel for 24 h (n = 3). (H) Western blot analysis of PARP, cleaved‐PARP, cleaved‐casp3, and aromatase in KGN cells treated with 1.5 μmol/L Hu‐17 in the presence or absence of 50 μmol/L pan‐caspase3 inhibitor Z‐VAD‐FMK (n = 3). All experiments were repeated on three separate occasions, n = 3 means 3 independent experiments. Data are presented as the mean ±SEM (A, B and E). Images represent Western blots (D, F, G and H). Statistical analyses were performed using paired t‐test (A) or ANOVA (B and E). * *p* < 0.05, ** *p* < 0.01, *** *p* < 0.001 vs control (0)

KGN cells, not other ovarian cell lines (A2790, SKOV3, and HO8910), are known to be steroidogenic ovarian granulosa‐like tumor cells that expresses abundant aromatase.^36^ Therefore, based on the SEA predictions, we used KGN cells to study the role of Hu‐17 in aromatase expression and estrogen synthesis. We found that estradiol levels were 52% lower in the culture medium of hGCs treated with 1.5 μmol/L Hu‐17 for 24 h compared with control (no‐treated) cells (Figure 3A). Additionally, estradiol levels were 79% lower in KGN cells treated with 1.5 μmol/L Hu‐17 than in control cells, although estradiol levels did not change when treated with 15 μmol/L cisplatin or 100 μmol/L paclitaxel treatment (Figure 3B). These experiments demonstrated that Hu‐17, but not first‐line chemotherapy drugs such as cisplatin and paclitaxel, could reduce estrogen biosynthesis, despite them all having similar effects on cell viability. Therefore, the decrease in estrogen production by Hu‐17 is not due to the decrease in the numbers of KGN cells.

Although Hu‐17 at various concentrations (0.034, 0.1028, 0.3086, 0.925, 2.77, 8.33, 25, and 75 μmol/L) had no detectable effect on aromatase activity when measured in vitro using a recombinant aromatase activity assay (Figure 3C), it decreased aromatase expression in both ovarian granulosa cells from OHSS patients (Figure 3D) and KGN cells (Figure 3E,F). KGN cells treated with 1.5 μmol/L Hu‐17 for 1, 2, 3, and 6 h had 25%, 50%, 26%, and 19% lower *CYP19A1* mRNA levels, respectively, compared with control cells (Figure 3E). Aromatase protein levels were also decreased in a time‐dependent manner in KGN cells treated with Hu‐17 for 2, 3, and 6 h (Figure 3F). These results suggested that Hu‐17 could inhibit estrogen production by decreasing aromatase protein expression rather than by directly blocking its activity.

In consistent with estrogen production, 1.5 μmol/L Hu‐17 also significantly decreased aromatase expression compared with control in KGN cells, but 15 μmol/L cisplatin or 100 μmol/L paclitaxel had no obvious effects (Figure 3G). To identify the mechanism by which Hu‐17 induces apoptosis, we simultaneously exposed KGN cells to 1.5 μmol/L Hu‐17 and 50 μmol/L Z‐VAD‐FMK, a universal caspase inhibitor, for 24 h. Z‐VAD‐FMK significantly reduced Hu‐17‐mediated apoptosis but did not block the Hu‐17‐induced reduction in aromatase expression (Figure 3H). These results suggested that Hu‐17 could inhibit estrogen production via the reduction of aromatase expression in an apoptosis‐independent manner in KGN cells.

### 3.4 Hu‐17 inhibits CREB‐1 phosphorylation in KGN cells

**FIGURE 4:**
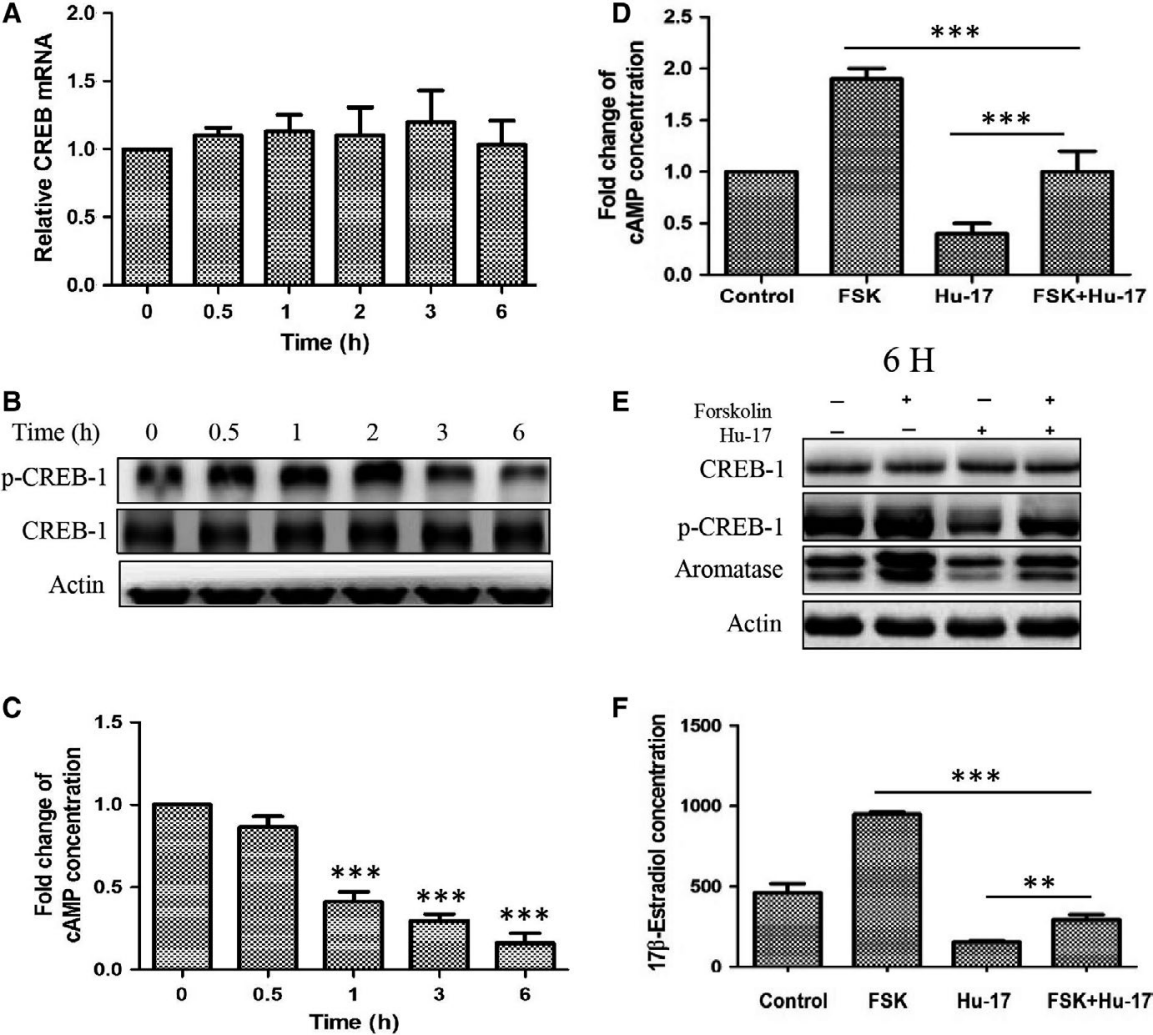
Involvement of cAMP/PKA/CREB pathway in Hu‐17‐induced inhibition of aromatase expression in KGN cells. (A and B) *CREB*‐*1* mRNA (A) CREB‐1 and phosphorylation of CREB‐1 protein expression (B) in KGN cells treated with 1.5 μmol/L Hu‐17 for 0.5, 1, 2, 3, and 6 h (n = 3). (C) cAMP level in KGN cells treated with 1.5 μmol/L Hu‐17 for 0.5, 1, 3 and 6 h (n = 3). (D, E and F) cAMP level (D), CREB‐1, phosphorylated of CREB‐1 and aromatase protein abundance (E), and 17β‐estradiol level (F) in KGN cells treated with 1.5 μmol/L Hu‐17 in the presence or absence of 50 μmol/L PKA agonist Forskolin for 6 h (n = 3). Data are presented as the mean ±SEM (A, C, D, and F). All experiments were repeated on three separate occasions, n = 3 means 3 independent experiments. Images represent Western blots (B and E). Statistical analyses were performed using ANOVA (A, C, D and F). **p < 0.01, ***p < 0.001 vs control (0)

The cAMP/PKA/CREB pathway is involved in the regulation of aromatase expression in ovarian granulosa cells.^10^ Therefore, we investigated the effect of Hu‐17 on cAMP/PKA/CREB pathway activation in KGN cells. Hu‐17 significantly inhibited CREB‐1 phosphorylation at 3 and 6 h (Figure 4B) but had no effect on *CREB*‐*1* mRNA and total protein in these cells (Figure 4A,B). Furthermore, 1.5 μmol/L Hu‐17 reduced intracellular cAMP levels at 1, 3, and 6 h in KGN cells (Figure 4C). Forskolin, an activator of adenylate cyclase, not only increased the basal cAMP levels but also reversed the Hu‐17‐induced decrease in intracellular cAMP in KGN cells (Figure 4D). Treatment with 1.5 μmol/L Hu‐17 also reversed the forskolin‐stimulated increase in CREB‐1 phosphorylation, aromatase expression (Figure 4E), and estrogen levels (Figure 4F). These results indicated that the cAMP pathway was involved in the downregulation of aromatase induced by Hu‐17 in KGN cells.

### 3.5 Hu‐17 causes changes in phosphorylation in KGN cells

**FIGURE 5:**
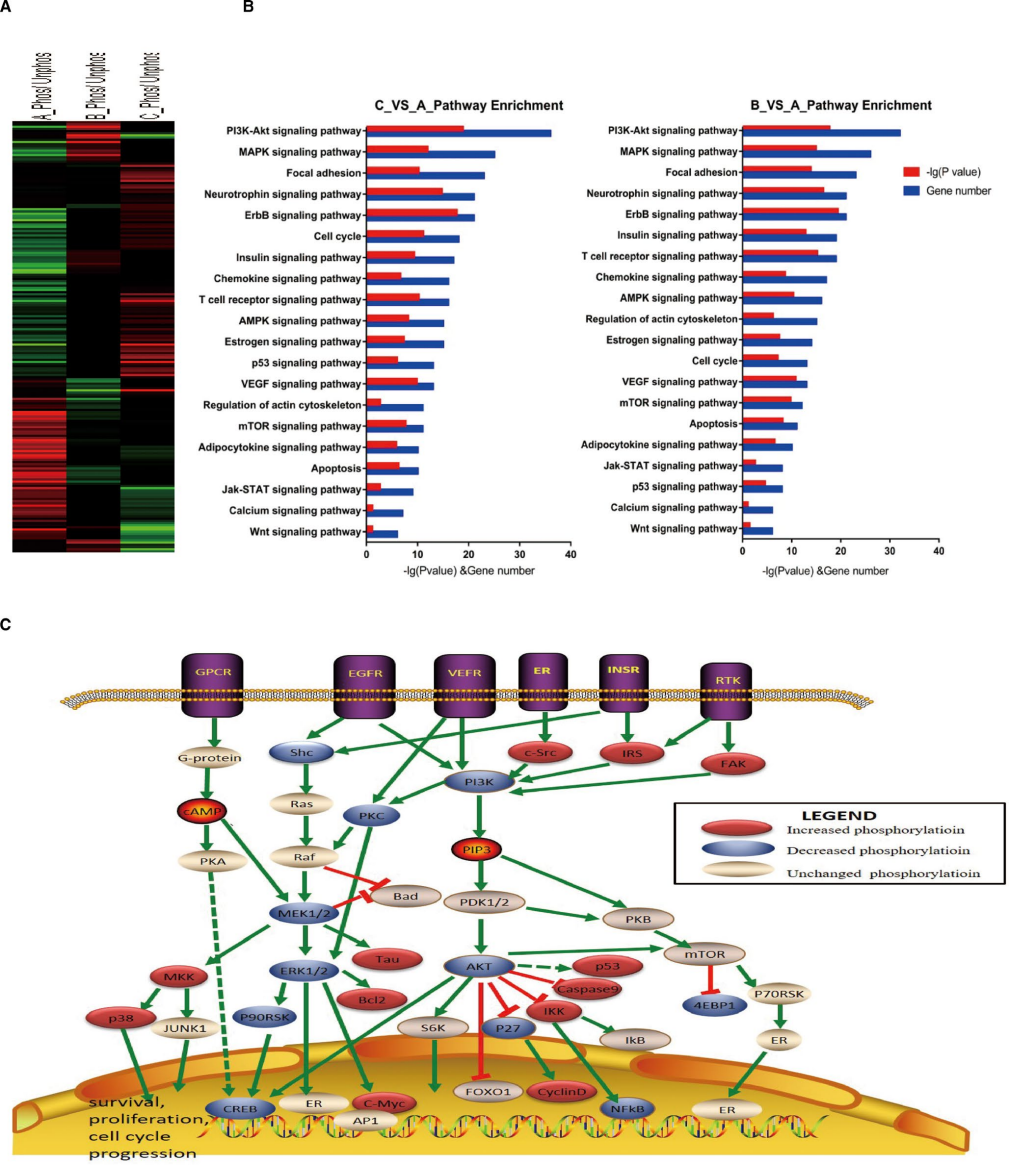
Phospho Explorer Antibody Array data of KGN cells treated with Hu‐17. (A) Heatmap of proteins differently phosphorylated proteins in KGN cells with Hu‐17 treatment. The data included in the heatmap is beyond cutoff line with the change fold is ≤77% or ≥130%. A is the control group, B is the Hu‐17 treated for 3 h group, C is the Hu‐17 treated for 6 h group. (B) Enrichment of signaling pathway according to KEGG by Hu‐17 treatment. (C) A proposed network display‐pathway analysis of key proteins differently phosphorylated proteins under Hu‐17 treatment in KGN cells. Proteins identified in this study regulated by Hu‐17 are represented in *red* (increased phosphorylation), *blue* (decreased phosphorylation) or *white* (unchanged phosphorylation)

Because SEA predicted possible protein phosphorylation changes with Hu‐17 treatment and we found that Hu‐17 inhibited CREB‐1 phosphorylation, we performed a phosphoproteomics‐based study using a phospho‐antibody microarray with 1318 antibodies to identify changes in protein phosphorylation with Hu‐17 treatment. After treatment with 1.5 μmol/L Hu‐17 for 0, 3, and 6 h, we identified proteins whose phosphorylation levels were increased or decreased by at least 1.3‐fold compared with controls. A total of 198 proteins including ERK (phospho‐Thr202/204), MEK (phospho‐Ser221/286), P90RSK (phospho‐Thr573), PI3 K (phospho‐Tyr467/Tyr199), and p53 (phospho‐Ser33) were identified (Figure 5A). KEGG pathway analysis yielded 20 significant pathways (Figure 5B), including PI3 K/AKT, mitogen‐activated protein kinase (MAPK), insulin, and estrogen signaling. The majority of the proteins regulated by Hu‐17 were involved in apoptosis, proliferation, and estrogen signaling (Figure 5C).

### 3.6 Hu‐17 blocks CREB‐1 phosphorylation via inhibition of ERK signaling

**FIGURE 6:**
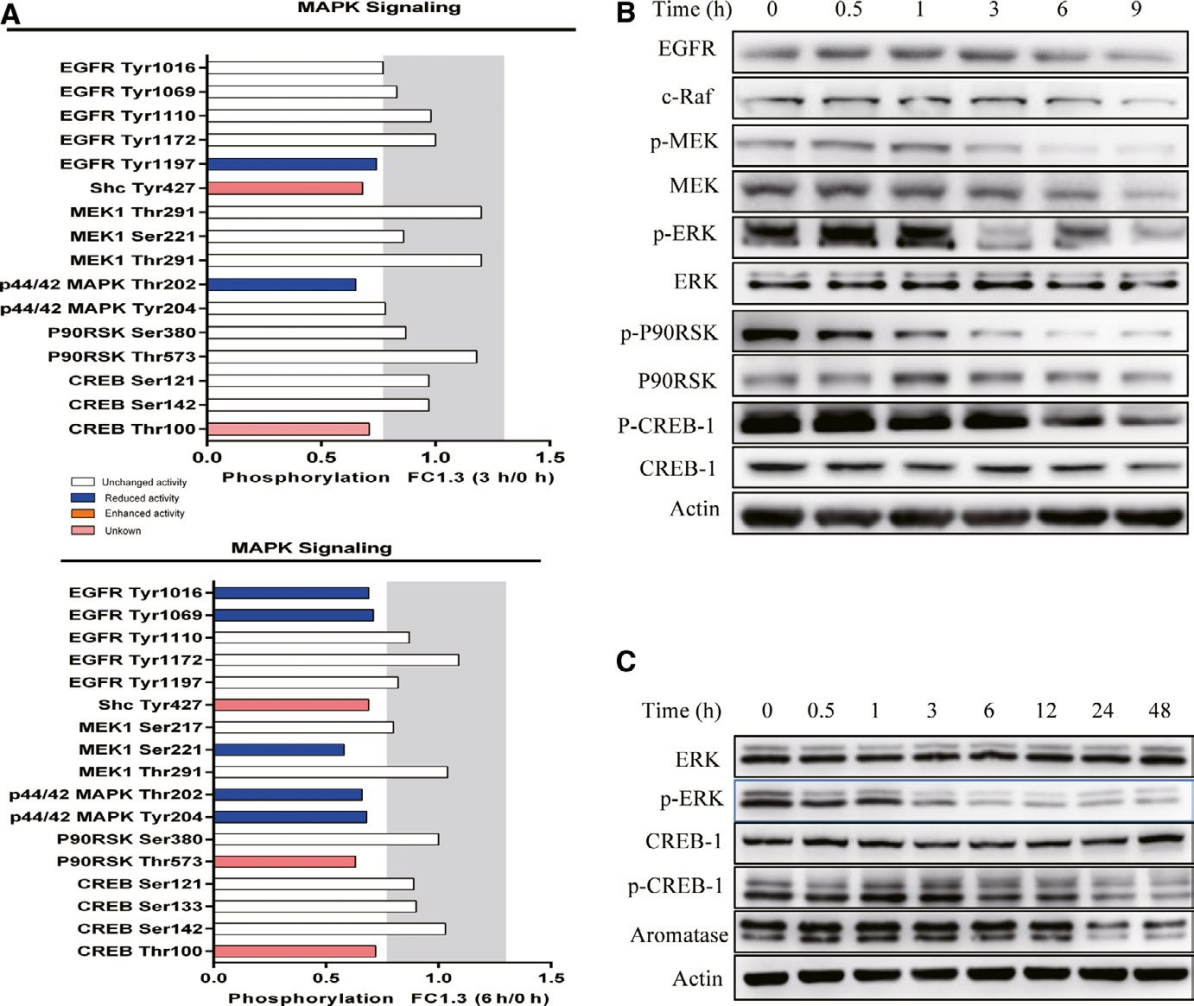
Involvement of ERK/CREB pathway in inhibition of aromatase expression in KGN cells treated with Hu‐17. (A) Fold changes of indicated phosphoproteins after normalization to total protein expression in KGN cells upon Hu‐17 treatment for 3 and 6 h. Gray‐colored areas indicate the defined induction/reduction boundaries (≤77%/≥130%). (B) Western blot analysis of EGFR, c‐raf, MEK, p‐MEK, ERK, p‐ERK, p90RSK, p‐p90RSK, CREB‐1, and p‐CREB‐1 in KGN cells after treatment with 1.5 μmol/L Hu‐17 for 0.5, 1, 3, 6 and 9 h (n = 3). (C) Western blot analysis of ERK, p‐ERK, CREB, p‐CREB‐1, and aromatase in KGN cells treated with 20 μmol/L PD98059 for 0.5, 1, 3, 6, 12, 24, and 48 h (n = 3). All experiments were repeated on three separate occasions, n = 3 means 3 independent experiments. Images represent Western blots (B and C)

In addition to PKA, the ERK and p38 kinase pathways are also involved in the regulation of CREB‐1 phosphorylation and aromatase expression.^37^, ^38^ Analysis of the phospho‐antibody microarray revealed that treatment with Hu‐17 inhibited the phosphorylation of several key proteins in the ERK pathway (Figure 6A), including ERK (phospho‐Thr202/204), MEK1 (phospho‐Tyr221), EGFR (phospho‐Thr1197), and P90RSK (phospho‐Thr573). We evaluated the phosphorylation of CREB‐1, ERK1/2, and upstream effectors P90RSK and MEK1 in KGN cells through selective confirmatory western blotting analysis to investigate whether Hu‐17 treatment affected CREB‐1 phosphorylation through the ERK pathway. As shown in Figure 6B, the phosphorylation of ERK, P90RSK, and MEK1 was decreased in KGN cells after Hu‐17 treatment for 3 h (Figure 6B). Moreover CREB‐1 phosphorylation and aromatase protein expression were decreased in KGN cells treated with the ERK inhibitor PD98059 for 24 h (Figure 6C). These data suggested that Hu‐17 acted through the ERK pathway to regulate aromatase expression in KGN cells.

### 3.7 Hu‐17 causes proteasome‐mediated degradation of aromatase in KGN cells

**FIGURE 7:**
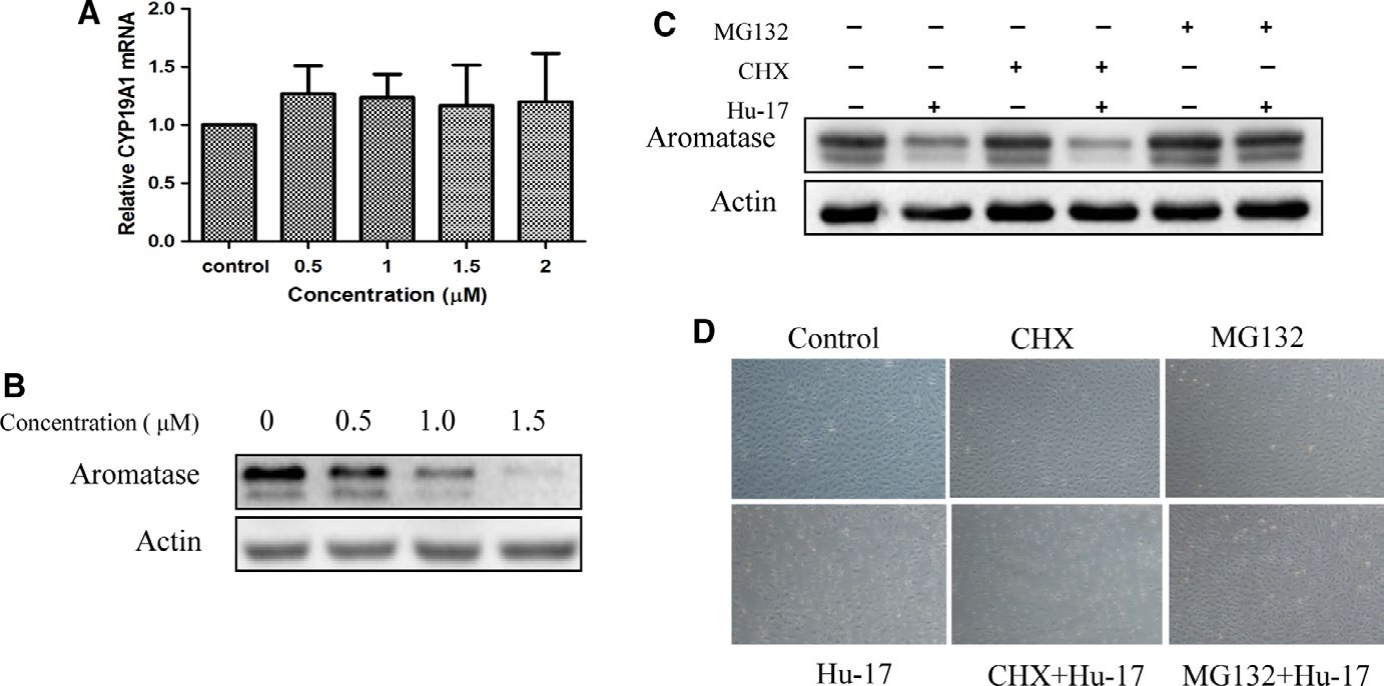
Hu‐17 induced degradation of aromatase protein via proteasome pathway in KGN cells. (A and B) *CYP19A1* mRNA expression (A) aromatase protein abundance (B) in KGN cells treated with 0.5, 1, 1.5, and 2 μmol/L Hu‐17 for 24 h (n = 3). (C and D) aromatase protein abundance (C) and morphological changes (D) in KGN cells treated with 1.5 μmol/L Hu‐17 in the presence or absence cycloheximide (CHX) (20 ng/mL) or MG132 (10 μmol/L) (n = 3). All experiments were repeated on three separate occasions, n = 3 means 3 independent experiments. Data are presented as the mean ±SEM (A). Images represent Western blots (B and C). Statistical analyses were performed using ANOVA (A). Morphological views (D) were magnified at 20× magnification

Interestingly, we found that Hu‐17 decreased *CYP19A1* mRNA at the earlier time points including 1, 2, 3, and 6 h but not at long time point such as 24 h (Figure 3E and Figure 7A), while it decreased aromatase protein level at both short time and long time points including 2, 3, 6, and 24 h (Figure 3F and Figure 7B) in KGN cells, suggesting that in addition to affect *CYP19A1* mRNA transcription, Hu‐17 may also could affect aromatase protein level on a post‐transcriptional level. Therefore, to better understand the mechanism of Hu‐17‐induced aromatase degradation, we treated KGN cells with Hu‐17 in the absence or presence with CHX (20 ng/mL), a de novo transcription inhibitor, as well as MG132 (10 μmol/L), a proteasome inhibitor, and found that CHX did not block Hu‐17‐induced reduction of aromatase protein while MG132 significantly attenuated the Hu‐17‐induced decreased in aromatase protein levels (Figure 7C), indicating that Hu‐17 didn't affect protein synthesis but induced aromatase protein degradation via proteasome pathway in KGN cells.

### 3.8 Aromatase protects KGN cells from apoptosis

**FIGURE 8:**
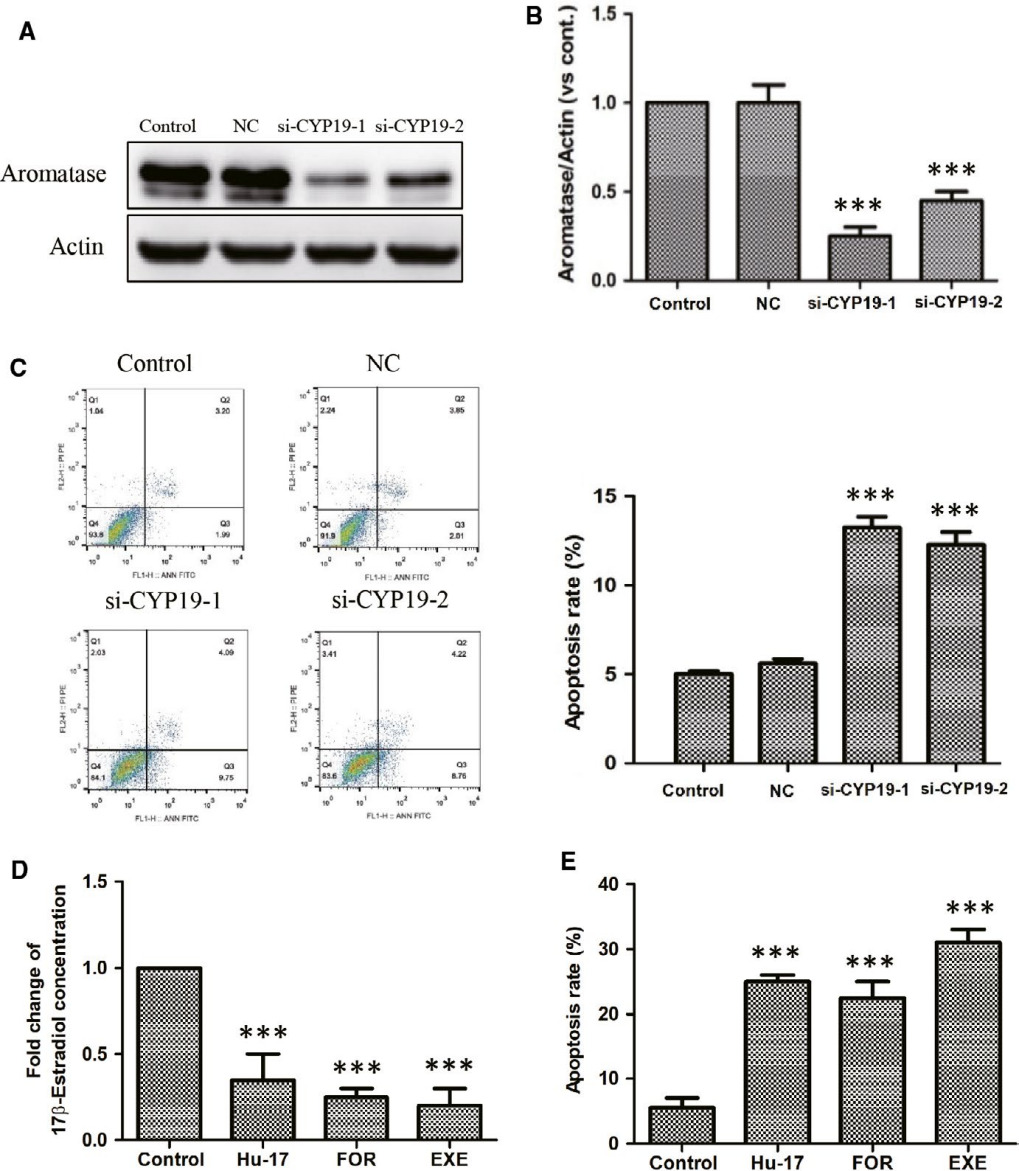
Involvement of aromatase abundance in the apoptosis of KGN cells. (A and B) The expression of *CYP19A1* mRNA (A) and aromatase expression (B) after *CYP19A1* gene knock‐down in KGN cells (n = 3). (C) Induction of apoptosis after *CYP19A1* gene knock‐down in KGN cells (n = 3). Apoptosis assays were performed using Annexin V‐FITC apoptosis detection kit by flow cytometry analysis. The left is the represent image of flow cytometry scatter plot, and the right represents the mean of 3 independent experiments. (D) Inhibition of 17β‐estradiol synthesis in KGN cells treated with 1.5 μmol/L Hu‐17, 100 nmol/L Formestane and 300 nmol/L exemestane for 24 h (n = 3). (E) Induction of apoptosis in KGN cells treated with 1.5 μmol/L Hu‐17, 100 nmol/L Formestane, and 300 nmol/L exemestane for 24 h, as evaluated through Annexin V‐specific antibody and propidium iodide (PI) staining, and followed by flow cytometry analysis (n = 3). All experiments were repeated on three separate occasions, n = 3 means 3 independent experiments. Images represent Western blots (A). Statistical analyses were performed using ANOVA. ***p < 0.001 vs control

Treating KGN cells with MG132 not only attenuated Hu‐17‐induced aromatase protein degradation, but also reversed apoptosis (Figure 7D). Therefore, to determine the relationship between aromatase stability and apoptosis, we explored the functional consequences of *CYP19A1* silencing on apoptosis in KGN cells, siRNA‐mediated knockdown of *CYP19A1* (Figure 8A,B) increased the proportion of apoptotic cells from 5.1% to 13.2% (Figure 8C). Consistently, treatment of KGN cells with aromatase inhibitor exemestane and formestane significantly decreased estrogen synthesis as well as the percentage of apoptosis (Figure 8D,E). These results suggested that aromatase abundance and estrogen level are involved in the Hu‐17‐induced KGN cells apoptosis.

### 3.9 Hu‐17 decreases proliferation and aromatase expression in breast cancer cells

**FIGURE 9:**
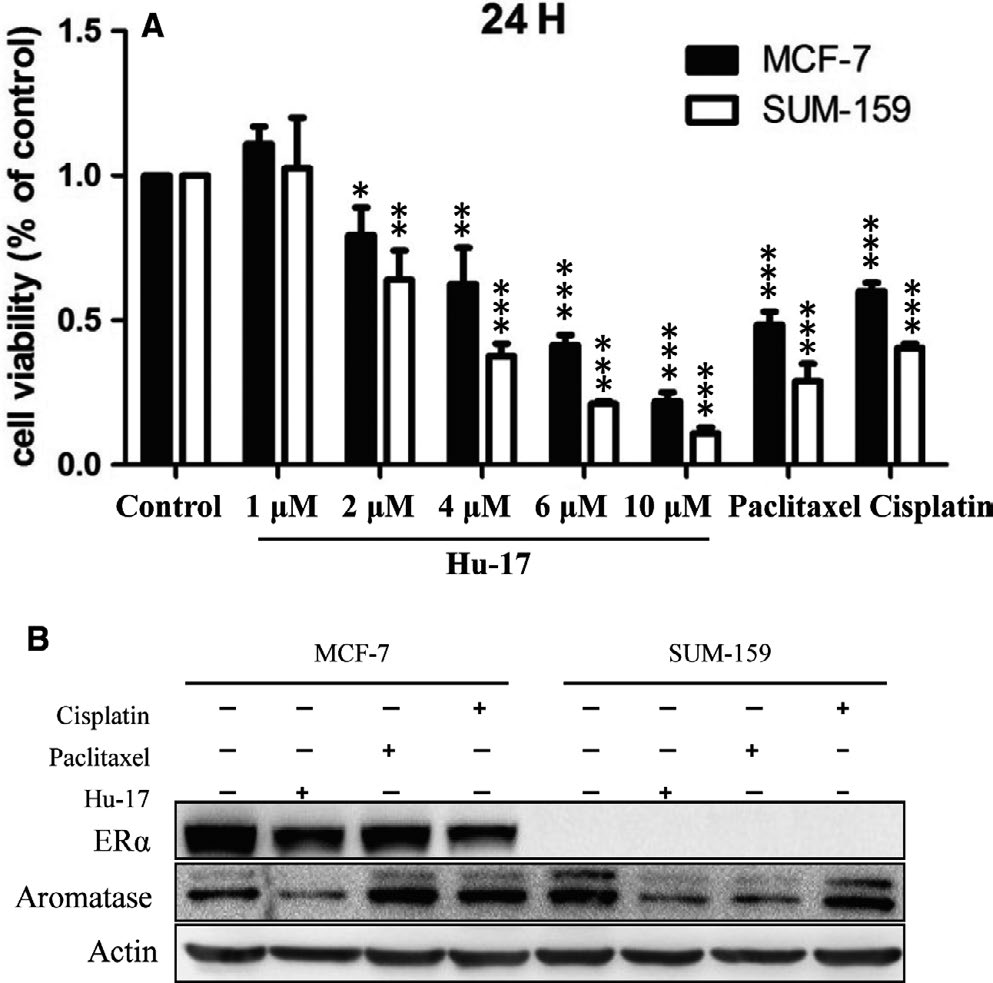
Hu‐17 inhibited cell proliferation and aromatase expression in breast cancer cells. (A) Cell viability of human breast cancer cells with 1, 2, 4, 6, and 10 μmol/L Hu‐17, 15 μmol/L Cisplatin, and 100 μmol/L Paclitaxel treatment for 24 h as assessed by MTT analysis (n = 3). (B) Protein levels of ERα and aromatase in human breast cancer cells (MCF‐7, SUM‐159) treated with Hu‐17, Cisplatin and Paclitaxel (n = 3). All experiments were repeated on three separate occasions, n = 3 means 3 independent experiments. Data are presented as the mean ±SEM (A). Images represent western blots (B). Statistical analyses were performed using ANOVA (A). *p < 0.05, **p < 0.01, ***p < 0.001 vs control (0)

Furthermore, to investigate the effects of Hu‐17 on aromatase expression in other gynecological malignancies, we used two breast cancer cell lines, MCF‐7 (ERα positive) and SUM‐159 (ERα negative) for an in vitro cell proliferation study. The viability was decreased in MCF‐7 and SUM‐159 cells treated with Hu‐17 at 2 μmol/L (81.2% and 76.3%, respectively), 4 μmol/L (76.1% and 47.5%, respectively), 6 μmol/L (48.2% and 20.4%, respectively), and 10 μmol/L (25.0% and 12.7%, respectively) for 24 h when compared with the control cells (Figure 9A). These effects in MCF‐7 and SUM‐159 cells were similar to those in KGN cells. Additionally, the aromatase protein level was decreased in both MCF‐7 cells treated with 2 μmol/L Hu‐17 and SUM‐159 cells treated with 4 μmol/L Hu‐17 (Figure 9B). These results suggested that Hu‐17 as an aromatase inhibitor might also have the potential to treat breast cancers.

## 4 DISCUSSION

In this study, we revealed the anticancer potential of Hu‐17 in ovarian human granulosa‐like tumor cells and breast cancer cells. Hu‐17 inhibited proliferation and induced apoptosis in KGN cells. Consistent with the predicted results of SEA, Hu‐17 inhibited aromatase expression and reduced estrogen levels in KGN cells and human ovarian granulosa cells at a concentration of 1.5 µmol/L. This concentration also led to an approximately 30% decrease in cell viability and a significant increase in apoptosis. Therefore, we chose 1.5 µmol/L for subsequent experiments. Based on the results of the SEA prediction, we used phosphokinase antibody arrays to identify signaling pathways affected by Hu‐17 treatment. Since the estrogen signaling pathway is significant in estrogen‐dependent gynecological tumors, we focused on the estrogen pathway and performed detailed mechanistic studies to explore new functions of Hu‐17. We also used cisplatin and paclitaxel to demonstrate that only Hu‐17, but not first‐line chemotherapy drugs, reduced aromatase expression and estrogen levels, despite the ability of all three drugs to induce apoptosis in KGN cells was similar. These results provided evidence that the reduction in aromatase expression and estrogen production by Hu‐17 is not due to the decreased numbers of KGN cells.

Chemotherapy drugs, including cisplatin and paclitaxel, are the current first‐line treatments for tumor of the ovary and breast.^41^ Cisplatin creates intrastrand and interstrand DNA crosslinks to block DNA replication and exerts its anticancer effects.^42^ Paclitaxel, a microtubule‐targeted drug, stimulates microtubule polymerization to block mitosis and induce apoptosis.^43^ Side effects of these drugs, such as neurotoxicity, myelosuppression, and drug resistance, are major obstacles in successful treatment outcomes. Therefore, endocrine therapy is an important adjuvant approach in preventing recurrences of estrogen‐dependent tumors. Further, endocrine therapy has more tissue specificity and less toxicity than chemotherapeutic regimens.^8^, ^44^, ^45^ Aromatase inhibitors are superior to tamoxifen and are the most widely used hormonal agents in the treatment of estrogen‐dependent tumors.^46^, ^47^, ^48^ Therefore, that Hu‐17 inhibits aromatase expression and reduces estrogen levels make it a promising candidate to treat estrogen‐dependent cancers.

Aromatase is a rate‐limiting enzyme of estrogen synthesis, and CREB‐1 is the key transcriptional factor in the regulation of aromatase in the ovaries. Aromatase expression is controlled by a promoter proximal to the start of translation in the ovary, which binds the transcription factors CREB‐1 and SF‐1.^49^, ^50^ CREB‐1 is phosphorylated and activated by PKA and other protein kinases, such as those in the ERK, p38, and PI3K pathways.^10^, ^37^, ^39^, ^40^ We found that Hu‐17 inhibited CREB‐1 phosphorylation without affecting total CREB‐1 expression. Additionally, Hu‐17 reduced intracellular cAMP levels. As intracellular cAMP homeostasis plays an important role in the activation of PKA in response to follicle stimulating hormone in ovarian granulosa cells, it appears that Hu‐17 regulates the cAMP pathway to decrease *CYP19A1* transcription in KGN cells. The expression of *CYP19A1* was suppressed by PD98059, a MEK/ERK pathway inhibitor, which is consistent with the results of previous studies in human primary granulosa luteal cells.^51^ However, some data show that only inhibitors of AKT/PI3K and p38 MAPK, not inhibitors of JNK (SP600125) and ERK (PD98059), significantly decreased aromatase expression in KGN cells.^52^ In our study, there was a significant difference in expression of *CYP19A1* mRNA before 24 h, while after 24 h, we could not observe these differences. The MEK/ERK pathway could regulate *CYP19A1* to an extent, although the PKA pathway played a vital role in *CYP19A1* expression. These results indicated that Hu‐17 reduced *CYP19A1* transcription by inhibiting CREB‐1 phosphorylation mainly through suppression of the PKA and ERK pathways, which is consistent with the results of the Phospho Explorer antibody microarray.

SEA also predicted that Hu‐17 had the potential to affect protein tyrosine phosphatases. Tyrosine‐specific protein phosphatases catalyze the removal of the phosphate group attached to a tyrosine residue. These enzymes are crucial regulatory components in signal transduction pathways, such as the MAPK pathway and cell cycle‐regulation. As such, they are important in the regulation of cell growth, proliferation, differentiation, and transformation.^53^, ^54^

In addition to decreasing *CYP19A1* mRNA expression, we also found that Hu‐17 could increase degradation of aromatase protein in KGN cells, which is similar to what we observed with exemestane, which promotes aromatase degradation through the ubiquitin/26S proteasome pathway without affecting *CYP19A1* mRNA in MCF‐7 cells.^55^ It will be interesting to investigate whether Hu‐17 and exemestane promote aromatase degradation by the same mechanism; however, no direct evidence was obtained. Therefore, the mechanism by which Hu‐17 regulates aromatase ubiquitination and proteasomal degradation needs to be further studied.

Although we demonstrated that the decrease in aromatase expression by Hu‐17 was not dependent on apoptosis, we found that knockdown of *CYP19A1* increased apoptosis in KGN cells, which suggested that the decrease in aromatase expression caused by Hu‐17 could result in apoptosis. TODA et al. found that mice lacking aromatase activity because of targeted disruption of *CYP19A1* exhibited anovulation and increased expression of pro‐apoptotic genes such as bax in the ArKO ovaries compared with their wild‐type siblings.^56^ Therefore, *CYP19A1* might have a role in regulating apoptosis. Furthermore, Hu‐17 had the same effects on the breast cancer cells lines MCF‐7 and SUM‐159. Moreover aromatase inhibitors are highly effective and less toxic than chemotherapy and are often offered to ER‐positive breast cancer patients to sustain a better quality of life.^57^

## 5 CONCLUSION

We demonstrate that the newly synthesized compound Hu‐17, as a potent inhibitor of estrogen biosynthesis, reduced *CYP19A1* expression and accelerated aromatase protein degradation in the human ovarian granulosa cancer cell line KGN. This research would draw attention to the study and development of new small‐molecule compounds as aromatase inhibitors. Therefore, Hu‐17 could serve as a potential treatment for estrogen‐dependent cancers as well as be helpful in designing new pharmaceutical tools for the prevention and treatment of estrogen‐dependent cancers.

## CONFLICT OF INTEREST

The authors declare no conflict of interest.

## AUTHORS’ CONTRIBUTIONS

YX and YZD conceived and designed the experiments. YHY synthesized and provided the chemical compound Hu‐17. YZD provided funding. YX, JSL, and HWW performed the experiments. YX and YZD analyzed the data and wrote the paper with the help of all the authors. All authors read and approved the final manuscript.

## Data Availability

All data were generated or analyzed during this study are included in this published article.

## References

[1] Wray N , Markovic M , Manderson L . Discourses of normality and difference: responses to diagnosis and treatment of gynaecological cancer of Australian women. Social Sci Med. 2007;64(11):2260–2271. 10.1016/j.socscimed.2007.02.034 17399878

[2] Ahmed N , Kadife E , Raza A , Short M , Jubinsky PT , Kannourakis G . Ovarian cancer, cancer stem cells and current treatment strategies: a potential role of magmas in the current treatment methods. Cells. 2020;9(3):719. 10.3390/cells9030719 PMC7140629 32183385

[3] Dong X , Men X , Zhang W , Lei P . Advances in tumor markers of ovarian cancer for early diagnosis. Indian J Cancer. 2014;51(Suppl 3):e72–e76. 25818738 10.4103/0019-509X.154049

[4] Futagami M , Yokoyama Y , Shimada M , et al. Contributions of the Japanese Gynecologic Oncology Group (JGOG) in improving the quality of life in women with gynecological malignancies. Curr Oncol Rep. 2017;19(4):25. 28303492 10.1007/s11912-017-0580-y

[5] Lin LL , Lakomy DS , Ning MS , Simpkins F , Jhingran A . Combining novel agents with radiotherapy for gynecologic malignancies: beyond the era of cisplatin. Int J Gynecol Cancer. 2020;30(4):409–423. 32193219 10.1136/ijgc-2020-001227

[6] Ghosh S . Cisplatin: the first metal based anticancer drug. Bioorg Chem. 2019;88:102925. 31003078 10.1016/j.bioorg.2019.102925

[7] Boyd LR , Muggia FM . Carboplatin/paclitaxel induction in ovarian cancer: the finer points. Oncology. 2018;32(8):418–420, 22–4. 30153322

[8] Levy A , Leynes C , Baig M , Chew SA . The application of biomaterials in the treatment of platinum‐resistant ovarian cancer. ChemMedChem. 2019;14(21):1810–1827. 31456347 10.1002/cmdc.201900450

[9] Bulun SE , Chen D , Lu M , et al. Aromatase excess in cancers of breast, endometrium and ovary. J Steroid Biochem Molecular Biol. 2007;106(1–5):81–96. 10.1016/j.jsbmb.2007.05.027 PMC2766613 17590327

[10] Stocco C . Aromatase expression in the ovary: hormonal and molecular regulation. Steroids. 2008;73(5):473–487. 18321551 10.1016/j.steroids.2008.01.017 PMC2365984

[11] Seidman AD . When to combine endocrine therapy with a new agent for hormone receptor‐positive metastatic breast cancer in postmenopausal women. Oncology. 2016;30(3):224–228. 26984215

[12] Zhu J , Zhao C , Kharman‐Biz A , et al. The atypical ubiquitin ligase RNF31 stabilizes estrogen receptor alpha and modulates estrogen‐stimulated breast cancer cell proliferation. Oncogene. 2014;33(34):4340–4351. 24441041 10.1038/onc.2013.573 PMC4141304

[13] Simpkins F , Garcia‐Soto A , Slingerland J . New insights on the role of hormonal therapy in ovarian cancer. Steroids. 2013;78(6):530–537. 23402742 10.1016/j.steroids.2013.01.008 PMC4551472

[14] Xu CY , Jiang ZN , Zhou Y , Li JJ , Huang LM . Estrogen receptor alpha roles in breast cancer chemoresistance. Asian Pacific J Cancer Prevention: APJCP. 2013;14(7):4049–4052. 10.7314/apjcp.2013.14.7.4049 23991950

[15] Balam FH , Ahmadi ZS , Ghorbani A . Inhibitory effect of chrysin on estrogen biosynthesis by suppression of enzyme aromatase (CYP19): a systematic review. Heliyon. 2020;6(3):e03557. 32181408 10.1016/j.heliyon.2020.e03557 PMC7063143

[16] Zeng C , Xu JN , Zhou Y , Yang HX , Zhou YF , Xue Q . C‐Jun NH2‐terminal kinase and p38 inhibition suppresses prostaglandin E2‐stimulated aromatase and estrogen receptor levels in human endometriosis. J Clin Endocrinol Metabolism. 2015;100(11):E1404–E1414. 10.1210/jc.2015-2031 26394174

[17] Howell A . Adjuvant aromatase inhibitors for breast cancer. Lancet. 2005;366(9484):431–433. 16084234 10.1016/S0140-6736(05)67036-5

[18] Jordan VC . Antiestrogens: clinical applications of pharmacology. J Soc Gynecologic Investigation. 2000;7(1 Suppl):S47–S48. 10.1016/s1071-5576(99)00059-3 10732329

[19] Johnston SR , Dowsett M . Aromatase inhibitors for breast cancer: lessons from the laboratory. Nature Rev Cancer. 2003;3(11):821–831. 14668813 10.1038/nrc1211

[20] Derks MGM , Blok EJ , Seynaeve C , et al. Adjuvant tamoxifen and exemestane in women with postmenopausal early breast cancer (TEAM): 10‐year follow‐up of a multicentre, open‐label, randomised, phase 3 trial. Lancet Oncol. 2017;18(9):1211–1220. 28732650 10.1016/S1470-2045(17)30419-9

[21] Bahrami N , Chang G , Kanaya N , et al. Changes in serum estrogenic activity during neoadjuvant therapy with letrozole and exemestane. J Steroid Biochem Molecular Biol. 2020;200:105641. 10.1016/j.jsbmb.2020.105641 32151708

[22] Goss PE , Ingle JN , Ales‐Martinez JE , et al. Exemestane for breast‐cancer prevention in postmenopausal women. New Eng J Med. 2011;364(25):2381–2391. 21639806 10.1056/NEJMoa1103507

[23] Aktas B , Sorkin M , Pusztai L , Hofstatter EW . Uptake of exemestane chemoprevention in postmenopausal women at increased risk for breast cancer. Eur J Cancer Prev. 2016;25(1):3–8. 25642790 10.1097/CEJ.0000000000000124 PMC4885537

[24] Foglietta J , Inno A , de Iuliis F , et al. Cardiotoxicity of aromatase inhibitors in breast cancer patients. Clin Breast Cancer. 2017;17(1):11–17. 27561703 10.1016/j.clbc.2016.07.003

[25] Figg WD 2nd , Cook K , Clarke R . Aromatase inhibitor plus ovarian suppression as adjuvant therapy in premenopausal women with breast cancer. Cancer Biol Ther. 2014;15(12):1586–1587. 25535893 10.4161/15384047.2014.972783 PMC4622589

[26] Di Liberto M , Svetaz L , Furlan RL , et al. Antifungal activity of saponin‐rich extracts of Phytolacca dioica and of the sapogenins obtained through hydrolysis. Natural Product Commun. 2010;5(7):1013–1018. 20734930

[27] Lounkine E , Keiser MJ , Whitebread S , et al. Large‐scale prediction and testing of drug activity on side‐effect targets. Nature. 2012;486(7403):361–367. 22722194 10.1038/nature11159 PMC3383642

[28] Keiser MJ , Setola V , Irwin JJ , et al. Predicting new molecular targets for known drugs. Nature. 2009;462(7270):175–181. 19881490 10.1038/nature08506 PMC2784146

[29] DeGraw AJ , Keiser MJ , Ochocki JD , Shoichet BK , Distefano MD . Prediction and evaluation of protein farnesyltransferase inhibition by commercial drugs. J Med Chem. 2010;53(6):2464–2471. 20180535 10.1021/jm901613f PMC2867455

[30] Zhang J , Liu J , Zhu K , et al. Effects of BMAL1‐SIRT1‐positive cycle on estrogen synthesis in human ovarian granulosa cells: an implicative role of BMAL1 in PCOS. Endocrine. 2016;53(2):574–584. 27117143 10.1007/s12020-016-0961-2

[31] Wang W , Guo C , Zhu P , et al. Phosphorylation of STAT3 mediates the induction of cyclooxygenase‐2 by cortisol in the human amnion at parturition. Sci Signal. 2015;8(400):ra106. 26508788 10.1126/scisignal.aac6151

[32] Zhu K , Li S , Liu J , Hong Y , Chen ZJ , Du Y . Role of RAB5A in FSHR‐mediated signal transduction in human granulosa cells. Reproduction. 2018;155(6):505–514. 29626103 10.1530/REP-18-0015

[33] Qian L , Liu Y , Xu Y , et al. Matrine derivative WM130 inhibits hepatocellular carcinoma by suppressing EGFR/ERK/MMP‐2 and PTEN/AKT signaling pathways. Cancer Lett. 2015;368(1):126–134. 26259512 10.1016/j.canlet.2015.07.035

[34] Zhai J , Liu J , Cheng X , et al. Zinc finger gene 217 (ZNF217) Promoted Ovarian Hyperstimulation Syndrome (OHSS) through Regulating E2 Synthesis and Inhibiting Thrombospondin‐1 (TSP‐1). Sci Rep. 2017;7(1):3245. 28607476 10.1038/s41598-017-03555-6 PMC5468349

[35] Eke I , Schneider L , Forster C , Zips D , Kunz‐Schughart LA , Cordes N . EGFR/JIP‐4/JNK2 signaling attenuates cetuximab‐mediated radiosensitization of squamous cell carcinoma cells. Cancer Res. 2013;73(1):297–306. 23074283 10.1158/0008-5472.CAN-12-2021

[36] Nishi Y , Yanase T , Mu Y , et al. Establishment and characterization of a steroidogenic human granulosa‐like tumor cell line, KGN, that expresses functional follicle‐stimulating hormone receptor. Endocrinology. 2001;142(1):437–445. 11145608 10.1210/endo.142.1.7862

[37] Martin LA , Farmer I , Johnston SR , Ali S , Marshall C , Dowsett M . Enhanced estrogen receptor (ER) alpha, ERBB2, and MAPK signal transduction pathways operate during the adaptation of MCF‐7 cells to long term estrogen deprivation. J Biol Chem. 2003;278(33):30458–30468. 12775708 10.1074/jbc.M305226200

[38] Gubbay O , Rae MT , McNeilly AS , Donadeu FX , Zeleznik AJ , Hillier SG . cAMP response element‐binding (CREB) signalling and ovarian surface epithelial cell survival. J Endocrinol. 2006;191(1):275–285. 17065410 10.1677/joe.1.06928

[39] Mayr B , Montminy M . Transcriptional regulation by the phosphorylation‐dependent factor CREB. Nat Rev Mol Cell Biol. 2001;2(8):599–609. 11483993 10.1038/35085068

[40] Hunzicker‐Dunn M , Maizels ET . FSH signaling pathways in immature granulosa cells that regulate target gene expression: branching out from protein kinase A. Cell Signal. 2006;18(9):1351–1359. 16616457 10.1016/j.cellsig.2006.02.011 PMC1564187

[41] McGuire WP 3rd , Markman M . Primary ovarian cancer chemotherapy: current standards of care. Br J Cancer. 2003;89(Suppl 3):S3–8. 10.1038/sj.bjc.6601494 PMC2750616 14661040

[42] Dai CH , Chen P , Li J , et al. Co‐inhibition of pol theta and HR genes efficiently synergize with cisplatin to suppress cisplatin‐resistant lung cancer cells survival. Oncotarget. 2016;7(40):65157–65170. 27533083 10.18632/oncotarget.11214 PMC5323145

[43] Jordan MA , Wilson L . Microtubules as a target for anticancer drugs. Nature Rev Cancer. 2004;4(4):253–265. 15057285 10.1038/nrc1317

[44] Dombernowsky P , Smith I , Falkson G , et al. Letrozole, a new oral aromatase inhibitor for advanced breast cancer: double‐blind randomized trial showing a dose effect and improved efficacy and tolerability compared with megestrol acetate. J Clin Oncol. 1998;16(2):453–461. 9469328 10.1200/JCO.1998.16.2.453

[45] Matikas A , Foukakis T , Michalakis I , Georgoulias V . Implementing neoadjuvant endocrine strategies in ER‐positive, HER2‐negative breast cancer. Expert Rev Anticancer Ther. 2017;17(4):319–326. 28128984 10.1080/14737140.2017.1288105

[46] Briest S , Davidson NE . Aromatase inhibitors for breast cancer. Rev Endocrine Metabolic Disorders. 2007;8(3):215–228. 10.1007/s11154-007-9039-z 17486453

[47] Campos SM . Aromatase inhibitors for breast cancer in postmenopausal women. Oncologist. 2004;9(2):126–136. 15047917 10.1634/theoncologist.9-2-126

[48] Ma CX , Reinert T , Chmielewska I , Ellis MJ . Mechanisms of aromatase inhibitor resistance. Nature Rev Cancer. 2015;15(5):261–275. 25907219 10.1038/nrc3920

[49] Lu DF , Yang LJ , Wang F , Zhang GL . Inhibitory effect of luteolin on estrogen biosynthesis in human ovarian granulosa cells by suppression of aromatase (CYP19). J Agricultural Food Chem. 2012;60(34):8411–8418. 10.1021/jf3022817 22838964

[50] Michael MD , Kilgore MW , Morohashi K , Simpson ER . Ad4BP/SF‐1 regulates cyclic AMP‐induced transcription from the proximal promoter (PII) of the human aromatase P450 (CYP19) gene in the ovary. J Biol Chem. 1995;270(22):13561–13566. 7768959 10.1074/jbc.270.22.13561

[51] Huang X , Jin J , Shen S , et al. Modulation of expression of 17‐Hydroxylase/17,20 lyase (CYP17) and P450 aromatase (CYP19) by inhibition of MEK1 in a human ovarian granulosa‐like tumor cell line. Gynecol Endocrinol. 2016;32(3):201–205. 26526982 10.3109/09513590.2015.1106470

[52] Guo J , Yuan Y , Lu D , et al. Two natural products, trans‐phytol and (22E)‐ergosta‐6,9,22‐triene‐3beta,5alpha,8alpha‐triol, inhibit the biosynthesis of estrogen in human ovarian granulosa cells by aromatase (CYP19). Toxicol Appl Pharmacol. 2014;279(1):23–32. 24853760 10.1016/j.taap.2014.05.008

[53] Demosthenous C , Han JJ , Hu G , Stenson M , Gupta M . Loss of function mutations in PTPN6 promote STAT3 deregulation via JAK3 kinase in diffuse large B‐cell lymphoma. Oncotarget. 2015;6(42):44703–44713. 26565811 10.18632/oncotarget.6300 PMC4792586

[54] Nai W , Threapleton D , Lu J , et al. Identification of novel genes and pathways in carotid atheroma using integrated bioinformatic methods. Sci Rep. 2016;6:18764. 26742467 10.1038/srep18764 PMC4705461

[55] Wang X , Chen S . Aromatase destabilizer: novel action of exemestane, a food and drug administration‐approved aromatase inhibitor. Cancer Res. 2006;66(21):10281–10286. 17079446 10.1158/0008-5472.CAN-06-2134

[56] Toda K , Takeda K , Okada T , et al. Targeted disruption of the aromatase P450 gene (Cyp19) in mice and their ovarian and uterine responses to 17beta‐oestradiol. J Endocrinol. 2001;170(1):99–111. 11431142 10.1677/joe.0.1700099

[57] Li F , Ye L , Lin SM , Leung LK . Dietary flavones and flavonones display differential effects on aromatase (CYP19) transcription in the breast cancer cells MCF‐7. Molecular Cellular Endocrinol. 2011;344(1–2):51–58. 10.1016/j.mce.2011.06.024 21741436

